# Books and Pamphlets Received

**Published:** 1887-03

**Authors:** 


### BOOKS AND PAMPHLETS RECEIVED.

*A Text-Book of Medicine fvr Students and Practitioners.* By Dr. Adolf Strumpell, formerly Professor and Director of the Medical Polyclinic at the University of Leipsic. Translated, by permission, from the second and third German editions by Hermann F. Vickery, A. B., M. D., Physician to Out-patients Massachusetts General Hospital ; Assistant in Clinical Medicine Harvard Medical School; Fellow of the Massachusetts Medical Society, etc. and Phillip Coombs Knapp, A. M., M. D., Physician to Out-patients with diseases of the Nervous system, Boston City Hospital ; Physician to the department for the diseases of the Nervous system, Boston Dispensary ; Fellow of the Massachusetts Medical Society, etc. With editorial notes by Frederick C. Shattuck, A. M., M. D., Visiting Physician to the Massachusetts General Hospital, and to the House of the Good Samaritan : Instructor in the Theory and Practice of Physic, Howard Medical School; Member of the Association of American Physicians, etc. With 111 illustrations. New York: D. Appleton & Co., 5 Bond street. 1887.

We have in the above a second and enlarged edition of a very able work on Practice, brought down to our latest advances in this interesting and important department. The book contains nearly 1,000 large octavo pages, is neatly and elegantly printed and beautifully illustrated.

Since the first edition much interesting matter on the pathology of the Nervous system has been added, and so the parts pertaining to typhoid fever, cholera and diphtheria have also received important additions. The work is well adapted to both students and practitioners. The general divisions under which the subjects are treated are: 1. Acute general infectious diseases ; 2. Diseases of the respiratory organs ; 3. Diseases of the circulatory system ; 4. Diseases of the digestive organs ; 5. Diseases of the nervous system ; 6. Diseases of the urinary organs ; 7. Diseases of the organs of locomotion ; lastly, Diseases affecting the blood and tissue metamorphosis.

Did our space permit, it would be interesting to refer to the treatment advised in certain affections. The following extract from his remarks on treatment of typhoid fever will attract attention, though may not be in all respects approved by American physicians : "If there is considerable diarrhœa we can give mistura gummosa (gum Arabic and sugar, each 15 parts ; water, 170 parts); tannin and subnitrate of bismuth, or small doses of opium. Constipation at the beginning of the disease is overcome by calomel. In latter stages we always try enemata first, to produce an operation. If this does not succeed, then we must employ rhubarb or castor oil. Considerable amounts of gas may be removed by introducing a long rectal tube."

"For internal hemorrhage, ice bags aad opium are relied upon. Every two hours the patient may take opium, half grain acetate of lead, one grain. Liquor ferri, 5 to 10 drops, every two hours, is often employed. Baths are not admissable when there is internal hemorrhage."

The remarks upon the treatment of pneumonia, though in the main judicious, gives to the American physician the impression that our Trans-Atlantic brethren are not fully up with the advances in the treatment of this affection. The rejection of veratrum, with the assertion that "we do not know a remedy which can in anyway exert a favorable influence upon the pneumonic process," will surprise many of our practitioners, especially those residing in the Southern States. With the views and treatment of other important maladies we are much pleased, and can recommend the work as, upon the whole, eminently useful and practical.

*A Text-Book of Pathological Anatomy and Pathogenesis.* By Ernest Ziegler, Professor of Pathological Anatomy in the University of Tubingen. Translated and edited for English students by Donald McAlister, M. A., M. B., Member of the Royal College of Physicians; Fellow and Medical Lecturer of St. John's College, Cambridge. Part II—Special Pathological Anatomy; Section 1, viii. New York: William Wood & Co. 1884.

The first section of this able and interesting work (which embraces 365 pages) treats of the blood and lymph; function of intravascular coagulation, or thromboses; changes in the quantity and composition of the blood; impurities in the blood; changes in the lymph, etc. The second section treats of the vascular mechanism; malformations and malpositions of the heart; endocarditis and myocarditis; infective granulomata; tumors and parasites of the heart; hypertrophy; hyperplasia, etc.; inflammations of the vessels; scleroris and atheroma; changes in the calibre of the vessels; rupture of the coats of the vessels; the lymphatics. Sec. III—Spleen and lymphatic glands. Section IV—The serous membranes. Section V—The skin. Section VI—The mucous membranes. Section VII—The alimentary tract. Section VIII—The liver and pancreas. Under these several heads numerous interesting topics are discussed. We commend the work as very interesting and useful to the progressive student of medical science.

*Manual of Operative Surgery.* By Joseph D. Bryant, M. D., Professor of Anatomy and Clinical Surgery, and Associate Professor of Orthopedic Surgery, Bellevue Hospital Medical College; Visiting Surgeon to Bellevue Hospital; Consulting Surgeon to the Bureau of Medical and Surgical Relief of Bellevue Hospital; Consulting Surgeon to the New York Lunatic Asylum and to the Northwestern Dispensary. With about 800 illustrations. New York: D. Appleton & Co. 1887.

A condensed and very comprehensive work of 530 large octavo pages. It has been brought out, as the author states, by the frequent request on the part of those whom it has been his pleasure to instruct in operative surgery during the past few years. Although very condensed, the work embraces the larger part of the more common and practical operations in surgery, in accordance with the latest and most approved methods, except the operations peculiar to the female sex, and the eye and ear, which could not be condensed in a work of the scope designed in the present volume. We are struck by the beauty and excellence of the illustrations, by the brevity and directness with which the author compasses the main and practical points in operative procedures, and by the absence of all that is useless and irrelevant in his descriptions. The work will doubtless be found eminently useful and acceptable to both student and practitioner.

*The Incidental Effects of Drugs;* a Pharmaceutical and Chemical Handbook. By Dr. L. Lewin, Assistant at the Pharmacological Institute of the University of Berlin. Translated by W. T. Alexander, M. D. New York: William Wood & Co.

We regard this as an important work, in that it treats of deviations from the normal or ordinary action of drugs. "A knowledge of these," says the author, "is of great importance to the physician, since they may, in a given case, shed light upon the cause of the unexpected phenomena which show themselves, and also furnish indications to guide him in his practical interference. Often the deviations from the usual effects of a drug are mistaken for a new phase of the disease, or for a complication to be met by an increased dose of the medicine.

Sometimes the patient has brought on the trouble by dosing himself, and the physician is called to treat the incidental symptoms caused by the remedy itself, and may unwittingly prescribe the same agent, thus aggravating to serious, if not fatal, extent the exciting trouble."

It is a work of 229 octavo pages, neatly printed, ably written. and containing much new and valuable information.

*An Epitome of the Newer Materia Medica. Standard Medicinal Products, and Fine Pharmaceutical Specialties.* Introduced and manufactured by Parke, Davis & Co. To which is added a complete property and dose list of all the fluid, solid and powdered extracts, German tinctures, normal liquids, and concentrations prepared by them ; together with a complete formula list of their sugar and gelatine coated pills. Fourth edition, revised and enlarged. 1886. Parke, Davis & Co., Detroit, Mich.

This is an excellent and convenient little work for the practitioner, wherein may be found much useful information adapted to his daily wants, and much pertaining to the new remedies which is often difficult to obtain at times when needed. The formula are very numerous, attractive and useful, and so also the dose list and properties of drugs.

*First Annual Report of the State Board of Health and Vital Statistics of the Commonwealth of Pennsylvania,* transmitted to the Governor, December 7, 1885.

This is a book of 360 octavo pages, kindly forwarded to us by the secretary, Benjamin Lee. A. M., M. D. It contains the by-laws, sanitary regulations, etc. of the Board ; the minutes of its sessions, and report and statistics from various sections of the State, with many beautiful maps illustrative of the topographical and geological features, the distribution of population, prevalence of disease, etc.

*The Doctor;* A Quarterly Journal of Medicine and Therapeutics. Peacock Chemical Company, Publishers, 307 Locust street, St. Louis, Mo.

*Monographia Syphilitica.* Illustrated. A Journal devoted to the Treatment of Diseases of the Blood and Skin by " Succus Alterans." Edited by Geo. W. McDade, M. D., Montgomery, Ala.

*Lactopeptine Medical Calendar.* By the New York Pharmacal Association. Containing, besides a well-arranged calendar, an abridged epitome of ancient and modern medicine and practice, and other interesting and useful reading matter.

*President's Address.* Tenth annual meeting of the Detroit Medical and Library Association. By C. J. Lundy, A. M., M. D.

